# Active surveillance of paratuberculosis in Alpine-dwelling red deer (*Cervus elaphus*)

**DOI:** 10.3389/fvets.2024.1303096

**Published:** 2024-01-25

**Authors:** Anita Filippi, Chiara Garbarino, Matteo Nava, Simone Russo, Joel Fernando Soares Filipe, Alessandro Bianchi, Luca Corlatti, Alessandro Gugiatti, Clelia Buccheri Pederzoli, Claudio Pigoli, Luca Pedrotti, Norma Arrigoni, Matteo Ricchi, Irene Bertoletti, Camilla Luzzago

**Affiliations:** ^1^Istituto Zooprofilattico Sperimentale della Lombardia e dell'Emilia Romagna, Sezione di Piacenza, National Reference Center for Paratuberculosis, Piacenza, Italy; ^2^Department of Veterinary Medicine and Animal Sciences, University of Milan, Lodi, Italy; ^3^Wildlife Health Lab, University of Milan, Lodi, Italy; ^4^Istituto Zooprofilattico Sperimentale della Lombardia e dell'Emilia Romagna, Sezione di Sondrio, Sondrio, Italy; ^5^Stelvio National Park—ERSAF Lombardia, Bormio, Italy; ^6^Wildlife Ecology and Management, University of Freiburg, Freiburg, Germany; ^7^Istituto Zooprofilattico Sperimentale della Lombardia e dell’Emilia Romagna, Sezione di Milano, Milan, Italy; ^8^WOAH Reference Laboratory for Paratuberculosis, Piacenza, Italy

**Keywords:** paratuberculosis, red deer, wild animal, prevalence, surveillance, Italy

## Abstract

Paratuberculosis (Johne’s disease) is a globally widespread infectious disease affecting domestic and wild ruminants, caused by *Mycobacterium avium subsp. paratuberculosis* (MAP). The bacterium is excreted in the feces and is characterized by high environmental resistance. The new Animal Health Law (Regulation EU 2016/429) on transmissible animal diseases, recently in force throughout the European Union, includes paratuberculosis within the diseases requiring surveillance in the EU, listing some domestic and wild Bovidae, Cervidae, and Camelidae as potential reservoirs. Taking advantage of a culling activity conducted in the Stelvio National Park (Italy), this study investigated MAP infection status of red deer (*Cervus elaphus*) between 2018 and 2022, and evaluated the probability of being MAP-positive with respect to individual and sampling-level variables. A total of 390 subjects were examined macroscopically and tested for MAP, using different diagnostic tools: IS900 qPCR, culture, histopathology, and serology. Twenty-three of them were found positive for MAP by at least one test, with an overall prevalence of 5.9% (95% CI 4.0–8.7), that, respectively, ranged from 12.4% in the first culling season to 2.0 and 2.1% in the 2019–2020 and 2021–2022 culling seasons. Quantitative PCR assay on ileocecal valve and mesenteric lymph nodes detected the highest number of MAP positive animals. The results of the study showed the increased probability of being MAP-positive with increasing age and that red deer with lower body mass values were more likely to be infected with MAP. Overall, the absence of signs of clinical paratuberculosis and gross lesions together with the low level of shedding witness early phases of the disease among the positive red deer and support an improvement of the paratuberculosis status of this population, as shown by the decreased prevalence of the disease over the years.

## 1 Introduction

Paratuberculosis is a worldwide distributed chronic intestinal infection caused by *Mycobacterium avium subsp. paratuberculosis* (MAP). MAP is a multi-host pathogen, highly resistant to severe environmental conditions (1). Many species are susceptible to MAP infection (2), but ruminants are the most susceptible, showing well-documented evidence of disease (3). By excreting MAP in feces, ruminants can contaminate the environment and infect other animals through fecal-oral route (4). Cattle, sheep, and goats are commonly affected, and wild ruminants, mainly cervids, are considered reservoirs, sharing MAP isolates with domestic livestock (5–8). Where domestic and wild species co-occur, interspecific transmission of disease may happen, also favored by environmental contamination by infected individuals (3, 9).

In Italy, the first reported case of paratuberculosis in wild ruminants was identified in red deer (*Cervus elaphus*) in the southern-Tyrolean part of the Stelvio National Park, in the Province of Bolzano (10). Afterward, the prevalence of MAP was estimated by molecular methods in red deer in several Alpine areas, showing values between 18.6 and 66.2% (11, 12), and an age-related pattern, with the highest prevalence in calves and yearlings (13). The Italian Ministry of Health has implemented paratuberculosis surveillance in cattle since 2013, recently extended to goat and sheep, on a voluntary basis, and a risk-based certification (14). The plan includes diagnostic screening, passive clinical surveillance on domestic ruminants, and adoption of biosecurity and management measures. Moreover, extensive livestock production, where domestic and wildlife animals interact, should consider both populations for disease control. The Animal Health Law (Regulation EU 2016/429) on transmissible animal diseases, recently in force throughout the European Union, includes paratuberculosis within the diseases that require surveillance in the EU, listing some domestic and wild Bovidae, Cervidae, and Camelidae as potential reservoirs (2).

The knowledge of the epidemiological role of wild animals is pivotal in order to progressively mitigate the risk of paratuberculosis infections in domestic animals. In Alpine areas, the risk of cross-transmission between domestic and wild ruminants is of particular concern during summer, as the interactions on pastures may facilitate the establishment of a multi-host system (15–17). The wildlife-livestock interactions have also been reported around supplementary feeding in wintertime (18). Taking advantage of a population of red deer subjected to a culling program in the Stelvio National Park (SNP), the aims of this study were: (i) to assess the temporal variation of MAP prevalence over three culling seasons and (ii) evaluate relationships between the probability of being MAP-positive and individual and sampling-level variables such as age, sex, body mass or individual conditions.

## 2 Materials and methods

### 2.1 Study area and population

The study area was located in the Lombardy sector of the SNP (Central Italian Alps) and ranges between 1,200 and 2,400 m a.s.l with an extension of approximately 2,600 ha, corresponding to the red deer wintering site. The red deer study population includes approximately 1,200 animals according to the annual counts, with a density of about 28 ind./km^2^ in the wintering site (19). Supplementary feeding was not applied in the study area. Other wild ruminants in the area include roe deer (*Capreolus capreolus*), Alpine chamois (*Rupicapra rupicapra*), and Alpine ibex (*Capra ibex*), which are present in very low densities and overlap with red deer in marginal areas. Furthermore, herds of cattle and small domestic ruminants share Alpine pastures with wild ruminants from mid-June until mid-September.

Given the impact on forest regeneration and biodiversity, since 2011 the Park has decided to reduce red deer density through culling in the wintering sites. The culling plan was authorized by the Italian Institute for Environmental Protection and Research (Prot. 48585/T-A25-Ispra).

### 2.2 Samples and data collection

Red deer were culled by authorized hunters in the cold season, between November and February, under the supervision of the Park Authority. The present study was carried out over three culling seasons (2018–2019, 2019–2020, and 2021–2022), as the COVID-19 pandemics caused the suspension of culling in 2020–2021. Considering that the culling activity was aimed at reducing the number of red deer inside the Park, culling has been focused on the young age classes and on females, rather than on mature males. However, both the age distribution and the sex distribution remain consistent across years, with the latter varied from a ratio of males-females of 1:1.7 in the first year to 1:1.3 in the third year (Supplementary Figures 1, 2). Culled red deer must underwent to veterinary inspection in an authorized game meat processing center, following EU Regulation for game meat hygiene, making it possible to sample individuals. Within a few hours after culling, animals were brought to the check point with entire gastrointestinal tract in the first culling season, whereas ileocecal valve and mesenteric lymph nodes were directly sampled in the field during the second and third culling seasons. Individual information including age, sex, body mass (the values refers to the weight of eviscerated animals) and kidney fat index (KFI) were collected to investigate the relationship with MAP status. A macroscopic inspection was performed on all individuals to detect signs of illness including poor body condition, diarrhea, and gross lesions. MAP status was investigated through: (i) tissue samples (361 samples of ileocecal valves and mesenteric lymph-nodes) stored at −20°C (additionally, a portion of tissue samples was stored in 10% formalin in 2018–2019 and 2019–2020); (ii) feces (*N* = 189) directly collected from rectum and stored at −20°C; (iii) blood serum (*N* = 315) from major vessels, obtained through centrifugation at 400 × *g* for 10 min of the whole blood and then stored at −20°C.

### 2.3 Real-time quantitative PCR on tissues and feces

To detect MAP-positive animals from tissue and feces, DNA was extracted from 25 mg of individual pools of ileocecal valves and mesenteric lymph-nodes (11) and from 3 ± 0.5 g of feces, as reported in Russo et al. (20). Quantitative PCR was performed on eluted DNA targeting IS900 sequence (20). Results were expressed by cycle of quantification (Cq) values, considering Cq values of two replicates of the same sample. In particular, a sample was considered positive if both replicates resulted ≤36 Cq, negative if both replicates were > 36 and inconclusive if one replicate resulted ≤36 and one >36. Analysis of inconclusive samples was repeated and if the inconclusive result was confirmed, the sample was considered negative.

### 2.4 Bacteriology

To further confirm the presence and viability of MAP circulating in the red deer population, samples positive to qPCR were submitted to culture. The bacteriological method was performed on 3 g of the individual pool of ileocecal valves and mesenteric lymph-nodes (*N* = 214), according to a method already described by Savi et al. (21). Briefly, the mucosa and lymph node parenchyma (if present) were decontaminated with Hexadecylpyridinium Chloride (HPC, 0.75% in sterile distilled water), washed with Phosphate Buffered Saline (PBS) and then incubated in Herrold’s egg yolk medium (HEYM), containing 2 mg of mycobactin J/L, supplemented with Chloramphenicol (30 mg/L) (HEYM/CAF) or with Nalidixic acid (50 mg/L), Vancomycin (50 mg/L) and sodium pyruvate (4 g/L) (HEYM/ANV). Results are reported considering the number of colony-forming units (CFU) and are expressed as: +, weakly positive (1–9 CFU/slant); ++, moderately positive (10–49 CFU/slant); +++, strongly positive (50–99 CFU/slant); and ++++, very strongly positive (> 99 CFU/slant). Suspected colonies were confirmed by qPCR targeting F57, Ziehl–Neelsen staining and Mycobactin dependency (7).

### 2.5 Histopathology

Formalin-fixed samples of the ileocecal valve and mesenteric lymph nodes were paraffin-embedded for histopathological examination. Two serial 3 μm thick sections were cut from each obtained paraffin block. One section was stained with hematoxylin–eosin. If microscopic lesions consistent with paratuberculosis infection were observed (22), the second section underwent Ziehl-Neelsen staining.

### 2.6 Serology

Sera were tested for MAP antibody presence through an Enzyme-Linked ImmunoSorbent Assay (ELISA) commercial kit (ID Screen® Paratuberculosis Indirect and ID Confirmation® Paratuberculosis Indirect, both ID VET, Montpellier, France) based on procedure steps indicated in the manufacturer’s protocol. The test employed has a specificity over 99%, thanks to a pre-adsorption step with *Mycobacterium phlei* and a screening test followed by a confirmation one. The test provides firstly a monocupola test, used to discriminate positive and inconclusive samples from negative ones; inconclusive (0.6 < S/P ratio < 0.7) and positive (S/P ≥ 0.7) samples to the screening test were then submitted to confirmatory test, where a bicupola kit was used in order to verify the specificity of the reaction. In more details, each sample resulted suspected positive or inconclusive to the first assay, was tested in duplicate; one duplicate was tested on a non-adsorbed well and the other on a well adsorbed with MAP antigen. Samples with a S/P ratio of 0.7 or above in the confirmation test were considered positive.

### 2.7 Statistical analysis

To investigate possible associations between MAP positivity and individual or sampling-level variables [sex, age class (calves, yearlings and adults of 2+ years), body mass, KFI and density], we used Generalized Linear Models (GLMs). The analyses were conducted with R (R Core Team 2022) in RStudio (Rstudio Team 2022). An individual was defined as MAP positive if at least one of the diagnostic tests (ELISA or PCR) resulted positive. MAP infection status [either 1 (positive) or 0 (negative)] was fitted as a binary response variable, while sex, age class, body mass, KFI, and density, were fitted as explanatory variables. Body mass and KFI were adjusted to the first day of culling by fitting quadratic linear models between any given value of the target variable and Julian date, within each year and for different age-classes (calves, yearlings, and adults) (23). Although deer are social animals organized in groups, the sexes stay mainly divided. This social behavior and population density may contribute to a possible association on the probability of MAP-positivity. The global model included the interaction of sex with individual variables, and was of the form:

$$\begin{array}{l} \mathrm{MAP}\;\mathrm{infection}\;\mathrm{status}\sim \mathrm{Year}+\mathrm{Sex}\times \\ \left(\mathrm{Age}\;\mathrm{class}+\mathrm{Body}\;\mathrm{mass}+KFI+\mathrm{Density}\right) \end{array}$$

All explanatory variables were scaled prior to model fitting, to make estimates comparable and avoid issues of collinearity in presence of interactions; density was not kept in the final model due to collinearity with other variables. The package “DHARMa” (24) was used for residual diagnostics. Starting from the global model, we selected a simpler structure using an AIC-based (Akaike Information Criterion) stepwise algorithm with the function “stepAIC” in the package “MASS” (25) and the marginal effects were plotted with the function “visreg” (26).

## 3 Results

Overall, out of the 390 individuals tested, 23 were found positive for MAP by at least one test, with a prevalence of 5.9% (95% CI 4.0–8.7), ranging from 12.4% (95% CI 8.0–18.8) in the first culling season to 2.0% (95% CI 0.5–7.0) and 2.1% (95% CI 0.7–5.9) in the 2019–2020 and 2021–2022 culling seasons, respectively. Apparent paratuberculosis prevalence in relation to culling season and other explanatory variables is reported in Table 1. MAP positivity was detected both in calves (≤1-year-old) and adults. During the inspection at the control center, no signs of clinical paratuberculosis or gross lesions at post-mortem examinations were detected in any culled red deer. Details about positive samples, diagnostic test results and type of biological specimen (tissues, feces, and serum) are summarized in Table 2. Overall, out of 23 MAP positive individuals, 19 were identified by qPCR assay carried out on tissue samples. The remaining four MAP positive animals were detected by serology, showing an S/P ratio > 1.7. None of the MAP positive animals showed positivity to all performed tests. Specifically, 15 out of 23 MAP positive samples were detected only by one test (12 by qPCR and 3 by serology), while the remaining animals resulted positive to both PCR and serology. Notably, qPCR assay performed on feces tested positive only in four out of 19 qPCR MAP positive on tissues. MAP isolation by cultural assay was achieved only in three samples out of 22 tested, all of them resulting weakly positive (1+, 1–9 CFU/slant) (Table 2); they were all collected from young animals.

**Table 1:** Paratuberculosis apparent prevalence according to culling season, age, sex, and type of sample of the Stelvio National Park.

Variable	Category	Positive/No. of tested animal (%)	Positive/No. of type of test (%)				
			qPCR tissue	qPCR feces	Culture tissue	Serology	Histopathology
Culling season	2018–2019	18/145 (12.4)	15/133 (11.3)	3/99 (3.0)	1/130 (0.8)	5/121 (4.1)	3/121 (2.5)
	2019–2020	2/100 (2.0)	1/84 (1.2)	0/33 (0)	1/84 (1.2)	1/62 (1.6)	0/54 (0)
	2021–2022	3/145 (2.1)	3/144 (2.1)	1/57 (1.8)	1/3^*^	1/132 (0.8)	n.p.^**^
Age	<1-year-old	4/126 (3.2)	4/112 (3.6)	1/64 (1.6)	1/70 (1.4)	0/103 (0)	0/54 (0)
	1 year old	3/62 (4.8)	3/60 (5.0)	0/27 (0)	2/30 (6.7)	1/49 (2.0)	1/27 (3.7)
	≥2 years old	16/202 (7.9)	12/189 (6.3)	3/98 (3.1)	0/114 (0)	6/163 (3.7)	2/94 (2.1)
Sex	Female	18/223 (8.1)	15/212 (7.1)	3/116 (2.6)	1/128 (0.8)	6/177 (3.4)	3/111 (2.7)
	Male	5/167 (3.0)	4/149 (2.7)	1/73 (1.4)	2/86 (2.3)	1/138 (0.7)	0/64 (0)
Total		23/390 (5.9)	19/361 (5.3)	4/189 (2.1)	3/214 (1.4)	7/315 (2.2)	3/175 (1.7)

^*^performed on qPCR positive on tissue. ^**^ not performed.

**Table 2:** Red deer testing positive to at least one diagnostic test.

ID	Culling data	Sex	Age (years)	qPCR tissue	qPCR feces	Culture^*^ tissue	Serology	Histopathology
1,067	2018-12-08	f	5.5	+	−	−	−	−
861	2018-12-15	f	2.5	+	+	−	+	+
959	2018-12-15	f	3.5	−	n.a.	−	+	−
1,047	2018-12-16	m	0.5	+	−	−	−	−
1,012	2018-12-18	f	12.5	+	−	−	−	−
1,147	2018-12-18	f	11.5	+	−	−	−	−
1,018	2018-12-20	m	0.5	+	−	−	−	−
986	2019-01-08	f	10.5	+	−	−	−	−
916	2019-01-22	f	0.5	+	−	−	−	−
996	2019-01-22	f	1.5	+	n.a.	−	−	−
1,049	2019-01-22	f	4.5	+	+	−	−	+
1,167	2019-01-22	f	2.5	+	−	−	−	−
1,168	2019-01-22	f	5.5	+	−	−	−	−
1,173	2019-01-22	f	1.5	+	−	+	+	+
1,174	2019-01-22	f	12.5	+	+	−	−	−
1,058	2019-02-09	f	7.5	−	−	−	+	−
1,126	2019-02-09	f	12.5	+	−	−	−	−
1,090	2019-02-12	f	9.5	−	−	−	+	−
1,234	2019-12-05	m	1.5	+	n.a.	+	n.a.	−
1,332	2020-01-16	m	2.5	n.a.	n.a.	n.a.	+	n.a.
1,231	2021-11-30	f	5.5	+	−	−	−	n.a.
1,118	2021-12-12	f	2.5	+	−	−	+	n.a.
1,225	2022-02-12	m	0.5	+	+	+	−	n.a.

n.a., specimen not available. ^*^The number of colony-forming units (CFU) and are expressed as: ^+^ (weakly positive: 1–9 CFU/slant); ^++^ (moderately positive: 10–49 CFU/slant); ^+++^ (strongly positive: 50–99 CFU/slant); and ^++++^ (very strongly positive: > 99 CFU/slant).

Considering histopathology, during the 2018–2019 season, out of 145 culled red deer, 121 underwent histopathological examination. In three animals, diffuse severe chronic granulomatous enterocolitis with Langhans-type multinucleated giant macrophages was found (Table 2); in the same subjects, granulomatous lesions were also found in the mesenteric lymph node. Ziehl-Neelsen staining revealed numerous intralesional acid-fast bacilli in these subjects. Notably, these three animals resulted positive to qPCR by tissues but showed a non-homogeneous trend to respect to the other assays employed. In the 2019–2020 season, 54 out of 100 culled red deer were subjected to histopathological investigations. None of the animals showed lesions consistent with paratubercular infection. No red deer culled during the 2021–2022 season underwent histopathological examination.

**Figure 1:**
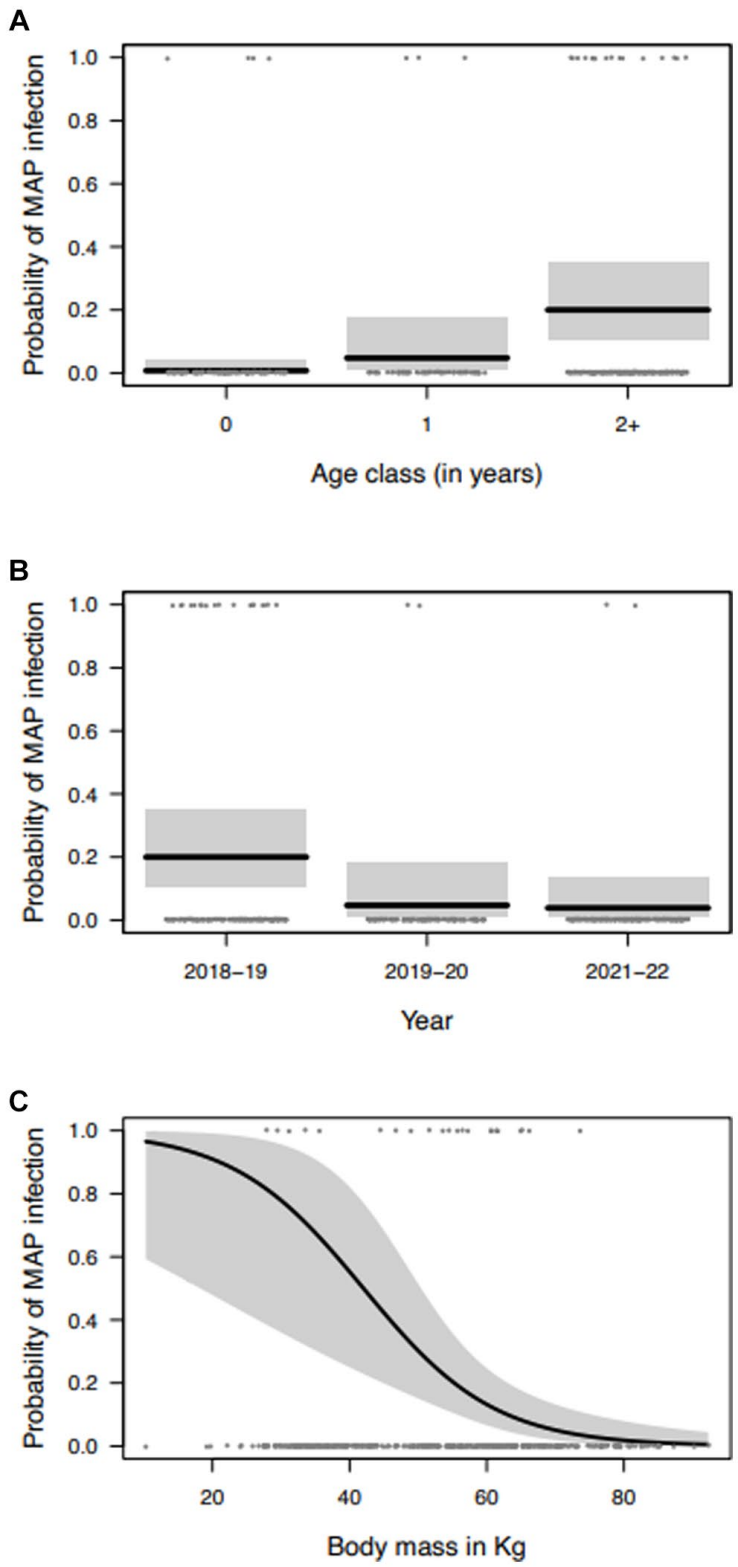
Marginal effects of the variables selected to explain the variation in the probability of MAP infection of red deer in the Stelvio National Park: **(A)** age class; **(B)** year of sampling; and **(C)** body mass (gray areas represent the 95% confidence intervals).

The model selection procedure performed to investigate possible associations between MAP positivity and collected variables returned a simpler model structure with the effects of year, sex, age class, body mass, and KFI and also the interaction of sex with body mass. However, this interaction was not significant and was excluded from the final model. Individual probability of being positive to MAP significantly increased with older age classes (Table 3; Figure 1A), with an estimated probability of 0.7% in calves, 4.7% in yearlings and 20% in adults, resulting in very high increases in the odds-ratio. With respect to year of sampling, probability of MAP positivity was significantly greater in 2018–2019 than in the remaining years with an estimated probability of 20% in 2018–2019, 4.7% 2019–2020, and 3.9% in 2021–2022 (Table 3; Figure 1B). Accordingly, there was an 80% decrease in the odds of being MAP positive from 2018–2019 to 2019–2020, and a decrease of 18% in the odds from 2019–2020 to 2021–2022. Body mass was negatively related to MAP positivity, as individuals with low body mass values were more likely to be infected with MAP than individuals with greater body masses, with a decrease of 82% in the odds of being MAP positive, per increase of 1 k in weight (Table 3; Figure 1C). Regarding KFI, the relation with MAP positivity was not statistically significant. Statistical analyses have been carried out also considering only the samples positive to tissue qPCR, but the results obtained did not differ from those obtained previously.

**Table 3:** Estimates of the final selected model.

Parameter	Log-Odds	SE	95% CI
(Intercept)	−4.59	0.82	[−6.34, −3.09]
Year [2019–20 vs. 2018–19]^*^	−1.62	0.78	[−3.51, −0.29]
Year [2021–22 vs. 2018–19]^*^	−1.83	0.68	[−3.39, −0.63]
Age class^2^ [1 vs. 0]^*^	1.9	0.91	[0.04, 3.73]
Age class^2^ [2+ vs. 0]^*^	3.5	0.98	[1.63, 5.54]
Body mass^*^	−1.69	0.49	[−2.73, −0.75]
KFI	0.48	0.25	[−0.02, 0.96]

The table shows for each parameter: the logarithm of the odds ratio (Log-Odds), the standard error (SE), and the 95% confidence intervals (95% CI). Age class in years: 0, 1, and 2 + . ^*^statistically significant.

## 4 Discussion

Surveillance for paratuberculosis has recently been introduced within the frame of European Animal Health Law in livestock and extended also to wild species. In this regard, paratuberculosis health status of red deer is important not only for the red deer population as such, but also for all species susceptible to the disease sharing the same habitat.

In the study area, the recovering of MAP field isolates with INMV1 profile has been described in culled red deer (11) and in environmental deer feces (27). This profile has been already described in Italian cattle population (7), supporting the hypothesis of possible transmission of MAP from cattle to red deer and vice versa. In this contest, the epidemiology of paratuberculosis was investigated in a red deer population in which the prevalence of paratuberculosis was previously assessed to be around 20% between 2011 and 2015 (11).

Our results showed an improvement of the infectious status of the population, with a decline of the prevalence over the years, until about 2% in the last culling seasons (2019–2020 and 2021–2022). These data are in line with the low apparent prevalence observed in the Lombardy region cow population, where Paratuberculosis is widespread (> 52% herds infected) but with low animal prevalence (1.3%) (28). In addition, despite MAP-positive animals were detected also in calves (≤1-year-old), the prevalence increased with age. This last result is in contrast with Galiero et al. (11) who reported a similar prevalence in young (up to 2 years old) and adult animals in the same area. Moreover, a previous investigation carried out in the Trentino sector of the Park, bordering our study area, showed higher MAP prevalence in young individuals (13).

A shift in the epidemiological pattern over the years, with a decreasing trend of MAP prevalence at the population level and in young animals, could be hypothesized. The results of the present study, specifically the increased probability of being MAP-positive with increasing age, supports this suggestion. On the other hand, the mortality induced by the disease itself, might have contributed to the infection decline. This phenomenon could have been exacerbated during the years of the study because of an increased snowfall (Supplementary Figure 3) which in turn, could have increased mortality due to winter starvation.

Notably, the qPCR assay performed on tissues (ileocecal valve and mesenteric lymph nodes) detected most of MAP positive individuals, while the test performed on feces detected MAP just in four out of 19 qPCR MAP positive on tissues. This result is in the line with what was reported in dairy herds (29), and could be related to the intermittent shedding of MAP in feces by infected individuals and to the amount of MAP excreted. The excretion varies according to the progression of the disease, being very low or null in the first phases of the infection (30). In fact, the histopathology performed in the present study showed lesions consistent with paratubercular infection in a low number of infected animals, of which only two were positive to qPCR by feces.

In relation to the high specificity and the low sensitivity of paratuberculosis tests used, we considered as positive an animal testing positive at least to one test. In our study we adopted a diagnostic parallel approach in order to increase the sensitivity of the detection. Despite the effort on histopathology, this method identified a low number of positive samples, probably also related to the limited size/area of tissue sampled. Moreover, our surveillance underlines the low sensitivity of serology and qPCR in feces, which are the methods most commonly used both in domestic and in wild ruminants (3, 29). Therefore, considering our results, for both active and passive surveillance, we recommend to analyze tissues by qPCR to define, with a higher accuracy, the paratuberculosis health status of wild population.

With respect to the correlation between the other variables investigated and the probability of MAP infection, the fact that red deer with low body mass values were more likely to be infected with MAP is consistent with the pathogenesis and the characteristics of the disease, which may lead to nutrients malabsorption and, consequently, to body mass loss. This index may reflect longer term effects, while KFI is a body condition metric that should reflect animal conditions in the short term. With respect to the results about KFI, close to the significance level, the sample size was limited, therefore any interpretation requires caution. Further data should be collected in order to correlate potential effects of MAP on KFI.

Overall, the absence of signs of clinical paratuberculosis and gross lesions together with the low level of shedding (MAP CFU), supports the possible presence of animals in early phases of the disease and with subclinical infections. Notably, at this stage, the disease could be still controlled by the immune system of the animals, without a progression of the disease to the clinical stage. In particular, these considerations, together with the decreasing trend of MAP prevalence in young animals, witness an improvement of health status of this population. The mortality induced by the disease itself, together with culling program, could have contributed to a reduction of MAP spreading in the red deer population of this area.

In addition, the adoption of national guidelines for the control of bovine paratuberculosis led to a general improvement of livestock health status (28) in the Lombardy region, with a reduced risk of spreading on pastures and transmission to wildlife.

In accordance with European Animal Health Law, it will be important to continue paratuberculosis surveillance both on wild and domestic ruminants in order to increase knowledge useful for the management of both populations, thus reducing the risk of infection in Alpine habitats.

## Data availability statement

The raw data supporting the conclusions of this article will be made available by the authors, without undue reservation.

## Ethics statement

Ethical approval was not required for the study involving animals in accordance with the local legislation and institutional requirements because no ethical approval was required for the present study and ethical statement is not applicable as sample collection from animals has been gathered after animals were culled for management purposes according to the official culling plan to reduce red deer density that has been authorized by Istituto Superiore per la Protezione e la Ricerca Ambientale (ISPRA), the Italian Ministry of Environment (Prot. 48585/T-A25-Ispra), in the Lombardy sector of the Park starting from 2011. Therefore, animals were not sacrificed for research purposes specific to this study.

## Author contributions

AF: Data curation, Formal Analysis, Writing – original draft. CG: Conceptualization, Funding acquisition, Project administration, Supervision, Writing – original draft. MN: Data curation, Formal Analysis, Investigation, Writing – review & editing. SR: Formal Analysis, Writing – review & editing. JS: Formal Analysis, Writing – review & editing. AB: Investigation, Writing – review & editing. LC: Data curation, Formal Analysis, Writing – review & editing. AG: Data curation, Investigation, Writing – review & editing. CB: Investigation, Writing – review & editing. CP: Formal Analysis, Writing – review & editing. LP: Funding acquisition, Supervision, Writing – review & editing. NA: Supervision, Writing – original draft. MR: Supervision, Writing – original draft. IB: Investigation, Writing – review & editing. CL: Conceptualization, Funding acquisition, Supervision, Writing – original draft.
