# Supplementary material for: Active surveillance of paratuberculosis in Alpine-dwelling red deer (Cervus elaphus)
Source: Front Vet Sci. 2024 Jan 25;11:1303096. doi: 10.3389/fvets.2024.1303096 (PMC10850319; doi:10.3389/fvets.2024.1303096)

Supplementary Material

1 Supplementary Figures and Tables

1.1 Supplementary Figures

Supplementary Figure 1. Histogram of the age distribution of the red deer culled divided by years of culling.

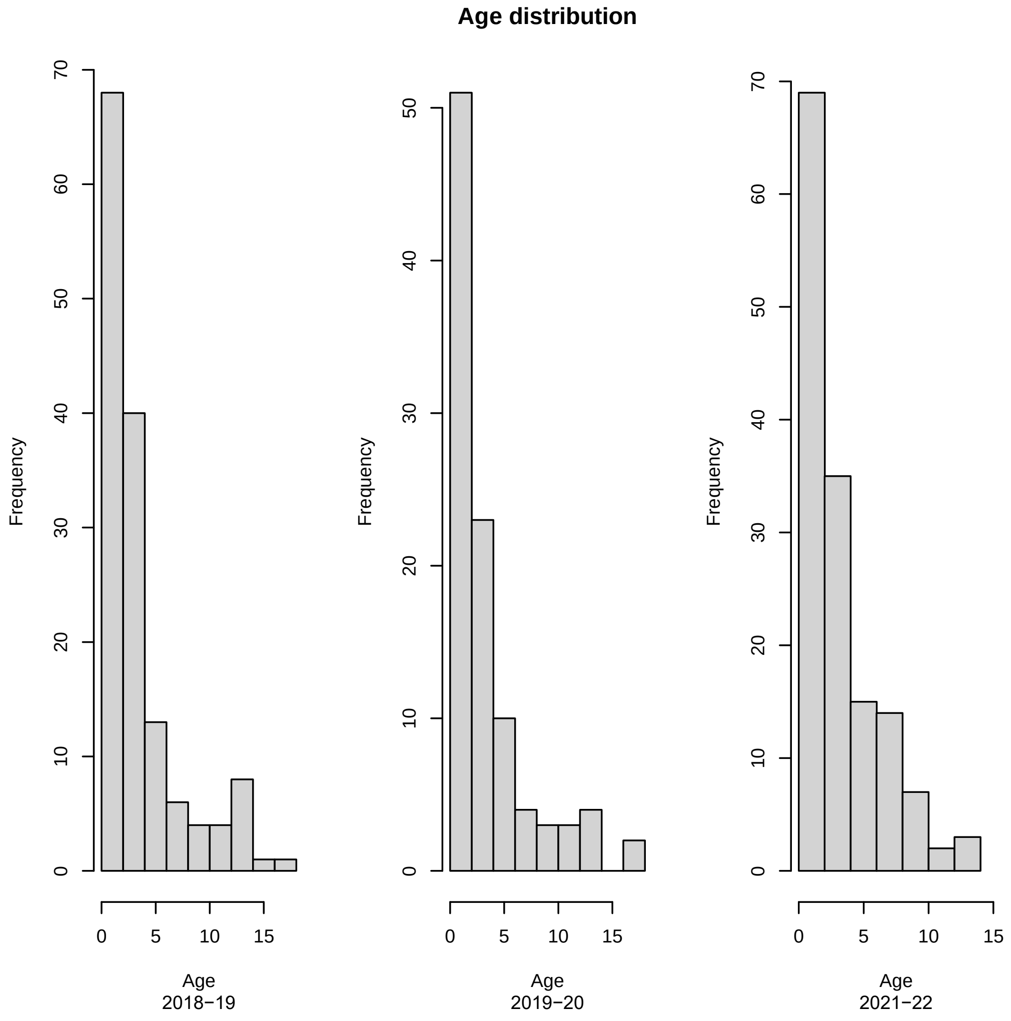

Supplementary Figure 2. Histogram of the sex distribution of the red deer culled divided by years of culling.

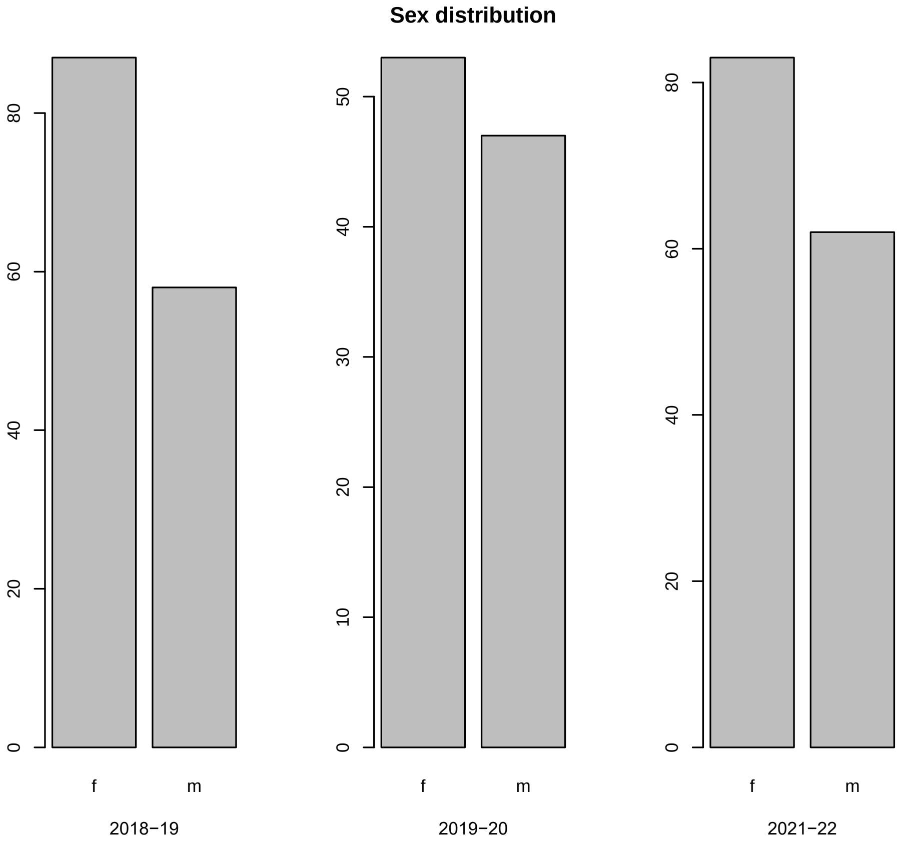

Supplementary Figure 3. Average snow depth in centimeters from 1st November to 30th April for each year from 2010. The weather station is close to the study area located at 2330 m.a.s.l. and the data are available from Arpa Lombardia (ARPA Lombardia - http://arpalombardia.it/temi-ambientali/meteo-e-clima/form-richiesta-dati/)

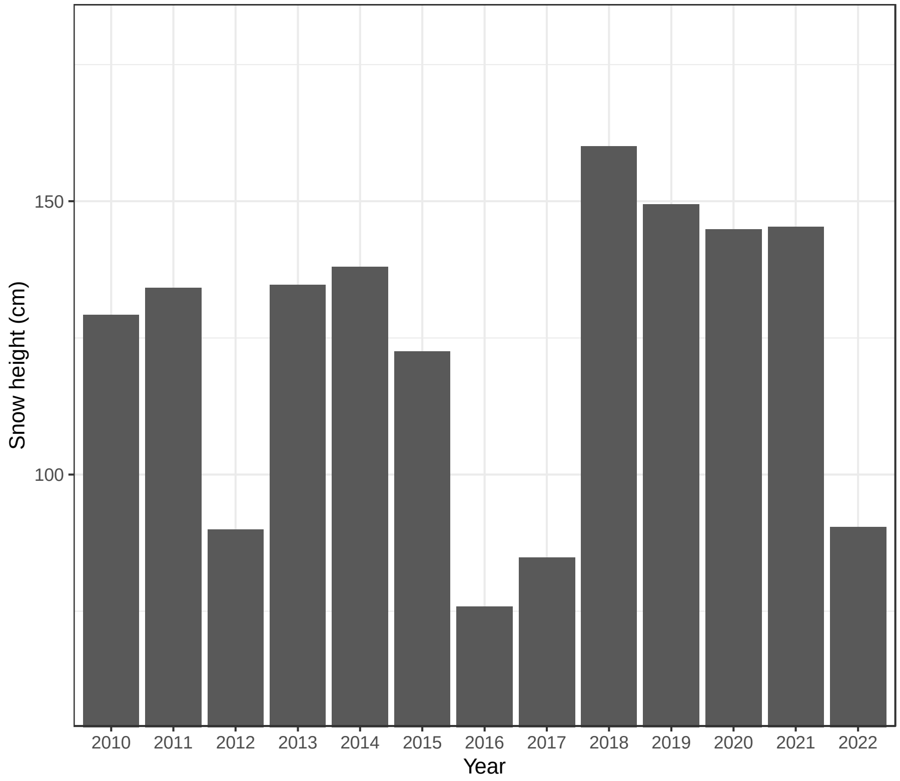

Supplement: Supplementary file 1 [file Data_Sheet_1.PDF]
